# Epstein–Barr virus transcription factor Zta acts through distal regulatory elements to directly control cellular gene expression

**DOI:** 10.1093/nar/gkv212

**Published:** 2015-03-16

**Authors:** Sharada Ramasubramanyan, Kay Osborn, Rajaei Al-Mohammad, Ijiel B. Naranjo Perez-Fernandez, Jianmin Zuo, Nicolae Balan, Anja Godfrey, Harshil Patel, Gordon Peters, Martin Rowe, Richard G. Jenner, Alison J. Sinclair

**Affiliations:** 1 School of Life Sciences, University of Sussex, Brighton BN1 9QG, UK; 2 School of Cancer Sciences, The University of Birmingham, Birmingham B15 2TT, UK; 3 Cancer Research UK London Research Institute, 44 Lincoln's Inn Fields, London WC2A 3LY, UK; 4 UCL Cancer Institute and MRC Centre for Medical Molecular Virology, Paul O'Gorman Building, University College London, London W1CE 6BT, UK

## Abstract

Lytic replication of the human gamma herpes virus Epstein-Barr virus (EBV) is an essential prerequisite for the spread of the virus. Differential regulation of a limited number of cellular genes has been reported in B-cells during the viral lytic replication cycle. We asked whether a viral bZIP transcription factor, Zta (BZLF1, ZEBRA, EB1), drives some of these changes. Using genome-wide chromatin immunoprecipitation coupled to next-generation DNA sequencing (ChIP-seq) we established a map of Zta interactions across the human genome. Using sensitive transcriptome analyses we identified 2263 cellular genes whose expression is significantly changed during the EBV lytic replication cycle. Zta binds 278 of the regulated genes and the distribution of binding sites shows that Zta binds mostly to sites that are distal to transcription start sites. This differs from the prevailing view that Zta activates viral genes by binding exclusively at promoter elements. We show that a synthetic Zta binding element confers Zta regulation at a distance and that distal Zta binding sites from cellular genes can confer Zta-mediated regulation on a heterologous promoter. This leads us to propose that Zta directly reprograms the expression of cellular genes through distal elements.

## INTRODUCTION

Epstein–Barr virus represents a serious health threat both in the developing world and in western economies and is a significant risk factor for Hodgkin's lymphoma (HL), Burkitt's lymphoma (BL) and nasopharyngeal carcinoma (NPC) (1–4). Primary infection with EBV is also responsible for the development of infectious mononucleosis (5). Infection of resting primary B-lymphocytes by EBV generates a population of cells that are effectively immortal. This represents the first step in the establishment of life-long viral latency *in vivo* and generates precursors that can develop into lymphoid malignancies (6,7).

EBV genes fall primarily into two groups, depending on their patterns of expression, and are classified as either latent or lytic (6,7). During latency, the majority of the viral genome is silenced and only a restricted set of genes is expressed (6,7). Once EBV lytic cycle replication is initiated, the lytic cycle genes are transcriptionally activated (8,9). Some lytic cycle genes are also expressed following infection of B-lymphocytes by EBV, but this does not result in a full lytic replication cycle (10–13) and has been termed a pre-latency phase (14).

The switch from viral latency to the lytic replication cycle is critical for virus spread. A key executioner of this process is the transcription and replication factor Zta (BZLF1, ZEBRA, EB1, Z) (9,15). This AP-1-like viral protein is a member of the bZIP family and interacts with a seven base-pair DNA sequence element termed Zta-response element (ZRE), for which at least 32 sequence variants are known (16,17). A sub-set of ZREs contain a CpG motif and are only recognized as ZREs when the DNA is methylated (11,18,19). DNA binding analyses across the EBV genome by ourselves and others revealed that Zta interacts extensively with viral promoters and with the viral origins of lytic replication (16,20). The current model of Zta function is that it acts at promoter-proximal regions to recruit RNA polymerase II and associated factors (15).

As well as regulating viral genes, Zta has the potential to alter the patterns of gene expression in the host cell. Zta is known to regulate the expression of a few cellular genes such as *FOS* (21), *E2F1* [23], *EGR1* (22,23), *IL8* (24), *IL10* (25) and *IL13* (26). Furthermore, attempts have been made to obtain a global map of cellular genes regulated during EBV replication using an EBV-positive BL cell line induced to initiate viral replication (27,28). However, as only a minority of the cells in a given population responds to the activating stimulus, this approach detected only the most highly activated genes.

Here, we undertook an unbiased genome-wide survey to map cellular genes that are bound by Zta. We then generated a cell system that allows enrichment of cells undergoing lytic cycle to perform sensitive transcriptome analyses of changes in expression of cellular genes during the lytic cycle. Integrating these data-sets led us to propose that Zta is able to regulate the expression of cellular genes through interactions with distal elements as well as through promoters, and we present further evidence to support this hypothesis.

## MATERIALS AND METHODS

### Cell lines

The Akata group I EBV-positive BL cell line (29), an EBV-immortalized lymphoblastoid cell line, LCL#3 (30) and an EBV-negative BL cell line, DG75 (31), were maintained in RPMI medium supplemented with 10% (v/v) fetal bovine serum, 100 units/ml penicillin, 100 μg/ml streptomycin and 2 mM l-glutamine (Life Technologies) at 37°C with 5% CO_2_. For EBV lytic induction, Akata cells were seeded in log phase growth at 5×10^5^cells/ml. After 24 h, the cells were concentrated to 2 × 10^6^ cells/ml and treated with 0.125% rabbit anti-human IgG (DAKO) or Dulbecco's phosphate buffered saline (DPBS) for the indicated times, in the presence or absence of acyclovir (Sigma) to capture both early and late lytic stages of viral replication (20). HONE1-EBV cells were maintained in the same medium and the presence of EBV within the cells was maintained by selection with 600 μg/ml G418. For EBV lytic replication cycle induction, HONE1-EBV cells were grown to 70% confluence and induced with 10 μM suberoylanilide hydroxamic acid (SAHA) for 48 h.

The expression vector pRTS-CD2-BZLF1, which drives expression of Zta, non-functional NGFR and GFP from a bi-directional doxycycline-regulated promoter and CD2 from a constitutive promoter or a control vector in which the BZLF1 sequences are cloned in the reverse orientation (32), were introduced into the Akata cell line by electroporation. Cells harboring the plasmids were physically enriched on the basis of CD2 expression, generating Akata-Zta and Akata control cells as described (32). Expression of the doxycycline-regulated promoter was induced by addition of 500 ng/ml doxycycline for 4–24 h and NGFR-expressing cells were isolated with anti-NGFR antibodies coupled to paramagnetic beads as described (32). Viable cell counts were assessed by trypan blue (Sigma) exclusion.

### FACS and protein analyses

GFP-positive cells were detected using multi-parameter fluorescent activated cell analysis (FACs) (Facs Canto-Beckton Dickinson). Intracellular staining of Zta was undertaken with the mouse monoclonal antibody BZ1 (33), with anti-mouse IgG coupled to Donkey F(ab’)2 as a secondary reagent (anti-mouse IgG -Alexa Fluor^®^ 647) and the FIX & PERM^®^ Cell Permeabilization Kit (LifeTechologies). Intracellular staining of VCA was undertaken with the mouse monoclonal antibody VCA-gp125 (clone L2 MAB8184, Millipore) and the same secondary reagent and conditions. Dual parameters of GFP and Alexa-fluor 647-coupled staining were collected and the double positive cells identified using BD FACSDiva™ Software (Beckton Dickinson).

Cells were harvested in sample buffer (4% SDS, 20% glycerol, 10% 2-mercaptoethanol, 0.004% bromophenol blue and 0.125 M Tris–HCl, pH 6.8.) and total proteins were fractionated by SDS-PAGE in a 12% gel (Novex). After transfer to nitrocellulose membrane, Zta and actin were detected by immunoblotting with the BZ1 mouse monoclonal antibody against Zta (33) and a rabbit antibody against β-actin (Sigma). Species-specific IR-labeled anti-mouse and anti-rabbit antibodies (Licor) were used in the second layer and the resulting signal was detected on an Odyssey Fc Imager and quantified using Odyssey Image Studio (Licor).

### Luciferase reporter assays

A 239 bp DNA element comprising 20 non-CpG ZREs (17) with a spacing element of 4–6 nucleotides between each was synthesized as complimentary oligonucleotides (Gene Strings—Life Technologies) and inserted between the SalI and BamH1 sites at the enhancer cloning site of the pGL3-enhancer reporter vector (Promega). Two minimal promoters, one from the cell CIITA gene (−214/+54) and the minimal promoter from the pGL4.23 reporter vector (Promega) (Supplementary Figure S4) were cloned upstream of the firefly luciferase sequences between the HindIII and Kpn1 restriction enzyme sites. The sequences of the cloned elements were verified (Eurofins). The viral BHLF1 promoter with the ZREs mutated was cloned upstream from the luciferase reporter gene in pCpGL luciferase reporter vector (34) and the Zta binding sites from the cellular FOSB gene and another region of cellular DNA were cloned upstream from it (Supplementary Figure S4). Zta binding sites ≥40kb downstream from the TSS of RASA3 were cloned into pGL3-enhancer reporter vector (Promega) and the minimal promoter from the pGL4.23 reporter vector was added (Promega) (Supplementary Figure S4).

Different DNA combinations comprising 5 μg of the relevant reporter construct and 5 μg of an expression vector for His-tagged Zta, or the corresponding empty vector control (35), were introduced into 1.2 × 10^7^ DG75 cells, an EBV-negative BL line (31), by electroporation with the Gene pulser II (Biorad). After 24 h, half of the cells were harvested into 250 μl of 1× passive lysis buffer (Promega), and luciferase activity was determined using the luciferase assay system (Promega). Light emission was measured in relative light units (RLU) using the GloMax multi detection system (Promega). The remaining cells were harvested into protein sample buffer and the levels of Zta were determined by immunoblotting (as described above). For each sample, relative promoter activity was determined from the ratio of luciferase activity to protein level and the assays were performed in triplicate.

### ChIP

Surface IgG on Akata cells was cross-linked by application of anti-IgG and 48 h later chromatin was prepared from 1 × 10^8^ cells. An additional sample was treated with acyclovir to halt viral replication prior to EBV genome replication. The chromatin was fixed by application of 1% (v/v) formaldehyde for 15 min at 20°C, extracted as described in Bark-Jones *et al*. (36) then sonicated on ice (10 × 10 s-pulses; 30% amplitude output) on a Branson model 250 Microtip at setting 5 (Sonics Vibacell). The chromatin was pre-cleared with protein A/G-sepharose bead slurry (Sigma) that had been pre-blocked in 0.5% (w/v) fraction V BSA (Sigma) in DPBS. 2% (v/v) of the pre-cleared extract was retained as the input control sample while the remainder was incubated with 10 μg of Zta-specific goat antibody (Santa Cruz # sc-17503) or control goat IgG for 1 h at 4°C as described in Ramasubramanyan *et al*. (20). The input control sample and the precipitated DNA were sequentially treated with 0.2 μg/ml RNAseA and 0.2 μg/ml Proteinase K and the DNA purified. Sequencing libraries were prepared using 10 ng of the input and ChIP DNA using a ChIP-seq sample preparation kit (Illumina) following the manufacturer's protocol, except that the library (150–350 bp fragments) was purified from the gel using a gel extraction kit (Qiagen) after PCR-amplification. The libraries were sequenced (single-end, 36 bp) using an Illumina Genome Analyzer IIx. Initial processing of sequencing images was carried out using the CASAVA pipeline. Base calling, and quality control statistics were performed using GOAT and Bustard modules. Sequence reads were aligned to the hg19 release of the human genome with ELAND. Bigwig files were generated by calculating tag density in 10-bp window, normalizing per million total reads and subtracting input signals using in-house R scripts. Peak calling (*P* < 10^−7^) was performed from a merged Bed file generated from the two ChIP-datasets using MACS (37), with a merged input Bed file acting as background (GSE57732). Distances between binding sites and the transcription start site annotated in RefSeq (38) were calculated. Genes within 2 kb of a peak were scored as promoter proximal. The Zta binding sequences associated with peaks were analyzed for common DNA-sequence motifs using MEME-ChIP (39,40). Matches with both *P* and *q* values of <1.0E−034 were selected and common microsatellite repeat sequences omitted. Functional gene enrichment analysis was undertaken using the DAVID bioinformatics resource at the National Institute of Allergy and Infectious Diseases (NIAID) (41,42).

ChIP was undertaken from chromatin generated from the spontaneously lytic LCL cell line LCL#3. Enrichment was quantified using Q-PCR spanning Zta binding peaks identified in the Akata cell line and flanking regions ∼2 kb 5′ or 3′ to them. The DNA sequences of each of the primers is shown in Table 1.

**Table 1.** Primers used for Q-PCR assays

	5′ flank	Zta peak	3′ flank
*SCIMP*		Forward primer CCCTCGTGCAATACTGTGAGA	Forward primer TTGCACAGCAAGTTCAAGCC
		Reverse primer ACAACTCATTCGCTCTGGGC	Reverse primer CTTTCTTGAAGGCAGATGGCAA
*BCL2A1*	Forward primer AGGAATTTGGCCTCCCAATCA	Forward primer TCTTGAGCTGGCTCACCTTG	Forward primer ACAGTGGTTACCTCTTGGGAGA
	Reverse primer TTTCTCCAGCGACCATGAGTT	Reverse primer AAACACAGCCTACGCACGAA	Reverse primer CCTGTGTTGAAACTCATGTTGGTA

### Reverse transcription and qPCR

Total RNA was prepared from Akata-Zta and Akata control cells 24 h after addition of doxycycline using RNAeasy Kit (Qiagen) and treated with RQ-DNAse (Promega) and quantitated using a Nanodrop spectrometer. First strand cDNA was prepared using random primers (Roche). Viral transcripts were amplified using absolute quantitation with Sybrgreen Go-Taq Q-PCR (Promega) with the following primers: BMLF1 (GACCGCTTCGAGTTCCAGAAT; ACTCTCCCGAACTAGCAGCAT and BDLF3 (TCGGTGGCAGTGATGTTCTG; TTCCAACGCATCCACCATCA). Host cell RNAs were also analyzed using TaqMan relative quantitation RT-PCR in a high-throughput format using Gene cards (Life Technologies, UK). Signals were normalized to GAPDH expression and the relative difference in the presence and absence of doxycycline determined. The fold change in RNA abundance was calculated together with the standard deviation from three independent cell enrichment experiments. The TaqMan primer sets were as follows: GAPDH-Hs99999905_m1; FOSB-Hs00171851_m1; SCIMP-Hs010294_m1; AN01-Hs00216121_m1; GDF2-Hs00211913_m1; FSCN1-Hs00602051_mH (Life Technologies, UK).

### RNA-Seq

Sequencing was performed on biological duplicates of Akata-Zta and Akata control samples on the Illumina HiSeq 2500 platform and generated ∼45 million 101-bp paired-end reads per sample. The data are deposited in GEO (GSE57732). Sequenced reads were mapped to RefSeq genes archived in the Illumina iGenomes resource (https://support.illumina.com/sequencing/sequencing_software/igenome.ilm using RSEM (version 1.2.4; (43)). An existing pipeline developed within the Trinity software package (http://trinityrnaseq.sourceforge.net/analysis/diff_expression_analysis.html) (44) was used to perform differential expression analysis with edgeR (version 3.01; http://www.bioconductor.org/packages/release/bioc/html/edgeR.html), which is available as part of the Bioconductor project developed within the R programming language (45). Genes with log CPM > 0, fold-changes above 2-fold and FDR<0.01 were judged to be differentially expressed. Genes with a total read count below 20 were excluded from further analysis. Views of the data are plotted using the Integrative Genomics Viewer (IGV) (46).

### DNA analysis

Total cell DNA was prepared from cells and the amounts of EBV genome relative to human genome determined using Q-PCR as described (47).

## RESULTS

### Candidate targets of Zta in the human genome

To determine whether Zta interacts with human genes during the switch from latency to lytic EBV replication, we undertook a genomic-scale analysis of Zta binding sites in Akata EBV-positive BL cells. Lytic replication was activated by engagement of the cell surface B-cell receptor (BCR) with anti-immunoglobulin. After crosslinking the proteins to DNA using formaldehyde, chromatin from the activated cells was immunoprecipitated with a previously validated Zta antibody (20,47) and the precipitated DNA was analyzed by massively parallel DNA sequencing (ChIP-seq). Significant regions of interaction between Zta and the cellular genome were identified using the Model-based Analysis of ChIP-Seq (MACS) algorithm (37). This identified 5020 Zta binding peaks (*P* ≤ 1×10^−7^). Using a motif-based sequence analysis tool (MEME-ChIP) (39,40) to search for enriched motifs we noted that the most frequent consensus sequences resembled the previously described non-CpG containing and CpG containing ZRE motifs from the EBV genome (16,17,20), suggesting that Zta directly binds to these sites in the human genome.

**Figure 1.**
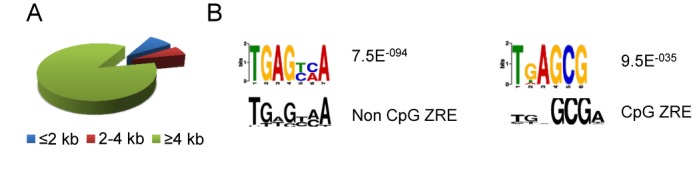
Zta binding across the human genome. ChIP-Seq was undertaken with an antibody against Zta protein using chromatin from Akata BL cells induced to enter EBV lytic replication cycle by cross-linking the B-cell receptor with anti-IgG. The DNA bound by Zta was aligned to the human genome and peaks of Zta binding were identified using model-based analysis of ChIP-seq (MACS). (**A**) The proportions of genes associated with proximal (<2 kb from TSS), intermediate (2–4 kb) and distal (>4 kb) Zta binding sites is shown. (**B**) The presence of common DNA sequence motifs within the Zta binding sites was determined using MEME-ChIP. The enriched motifs are colored together with the significance of the enrichment. The related ZRE motif is shown below each in black (17).

Although some of the Zta binding sites (14%) were close to the annotated transcriptional start sites (TSS) of cellular genes (Figure 1B and Supplementary Table 1), analogous to the situation observed for the interaction of Zta with viral genes, the majority of the Zta binding sites (86%) were more distant (≥4 kb from the nearest TSS). By assigning each of the Zta peaks to the closest TSS, irrespective of distance, we identified 2395 cellular genes as potential candidates for regulation by Zta.

**Figure 2.**
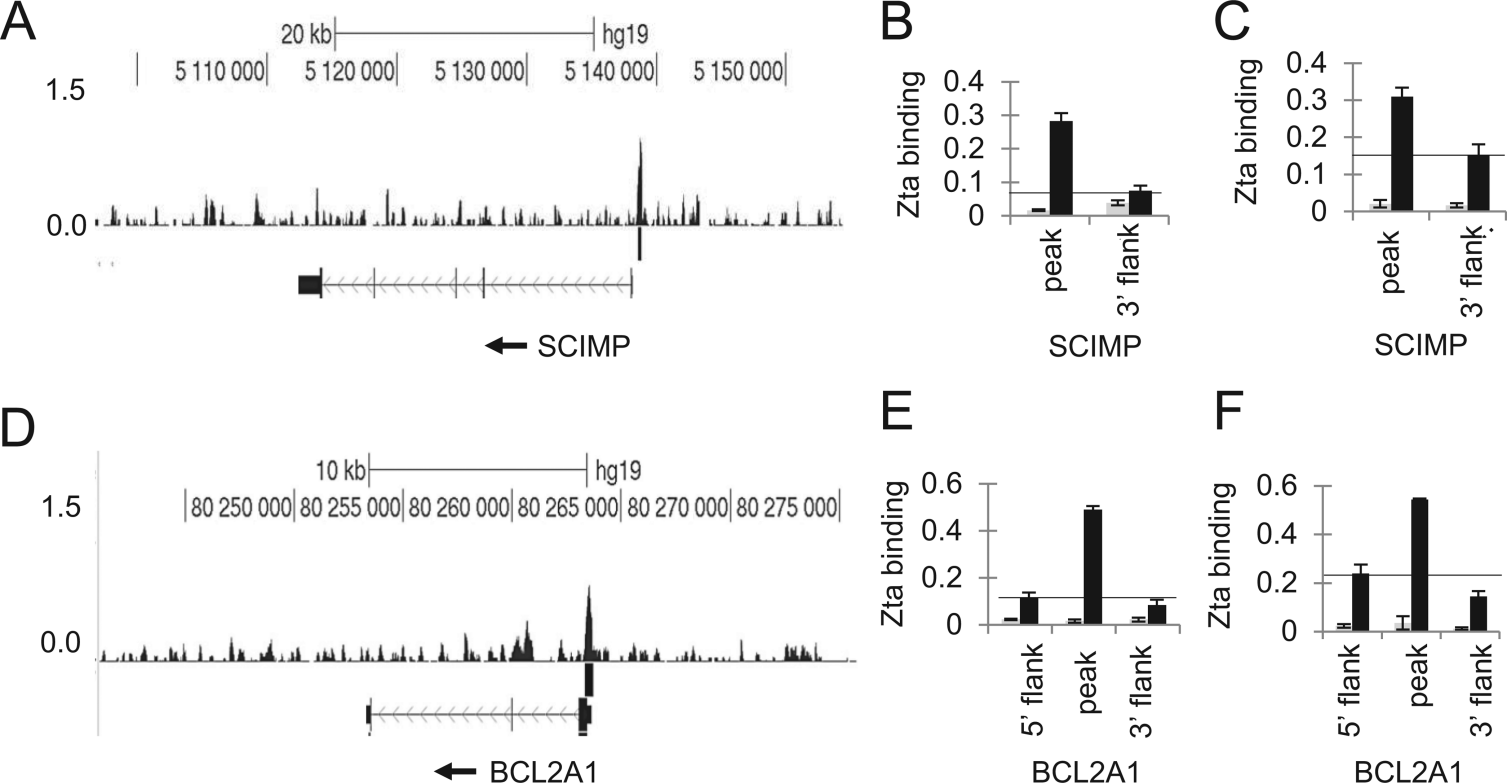
Comparison of Zta binding patterns in BL, LCL and NPC cells. (**A**) Examples of ChIP-seq data from Akata cells aligned to the human genome surrounding the *SCIMP* locus (chr17:5 096 004–5 154 368, hg19) is shown using the UCSC genome browser. The depth of sequencing reads from Zta-enriched DNA is plotted per million background-subtracted total reads. The scale bar and genome location are shown, together with the Zta binding profile. Beneath this are the peaks of Zta binding identified using MACs (*P* < 10^−7^), with the RefSeq gene mapping shown below that. (**B**) The presence of Zta binding in LCL#3 is shown at the *SCIMP* locus using Q-PCR with primers corresponding to the Zta binding sites (peak) and a flanking region 2 kb distant (flank). The data from triplicate analyses show the percentage of binding relative to input chromatin. The DNA binding associated with Zta is shown in black and the control antibody in gray. A horizontal line marks the baseline for the assay. (**C**) The presence of Zta binding in HONE1-EBV at the *SCIMP* locus is shown (as in B). (**D**) Examples of ChIP-seq data from Akata cells aligned to the human genome surrounding the *BCL2A1* locus (chr15:80 241 085–80 275 791, hg19) is shown using the UCSC genome browser as in (A). (**E**). The presence of Zta binding in LCL#3 is shown at the *BCL2A1* locus using Q-PCR with primers corresponding to the binding sites and to two flanking regions 2 kb distant either side of the site as in (B). (**F**) The presence of Zta binding in HONE1-EBV cells at the *BCL2A1* locus is shown.

To validate the ChIP-seq, we chose representative examples of cellular genes associated with Zta binding sites, based on the patterns of Zta binding and amenability to analysis by Q-PCR using PCR primer sets that would discriminate enhanced binding within the peak region relative to the adjacent DNA. We also determined whether Zta binding occurs in different cell types in which EBV undergoes lytic replication: the LCL#3 lymphoblastoid cell line (30) and HONE1-EBV, a cell line derived from a nasopharyngeal carcinoma (48). Examples of the analysis of Zta binding at the *SCIMP* and *BCL2A1* genes are shown in Figure 2A and D. In both cases, Zta binding was enriched within the peak region identified by ChIP-seq in Akata cells undergoing lytic replication (Figure 2B, C, E and F). This demonstrates that the specific interaction between Zta and these genes occurs in three different cell backgrounds.

### Changes in the cell transcriptome during EBV lytic cycle

**Figure 3.**
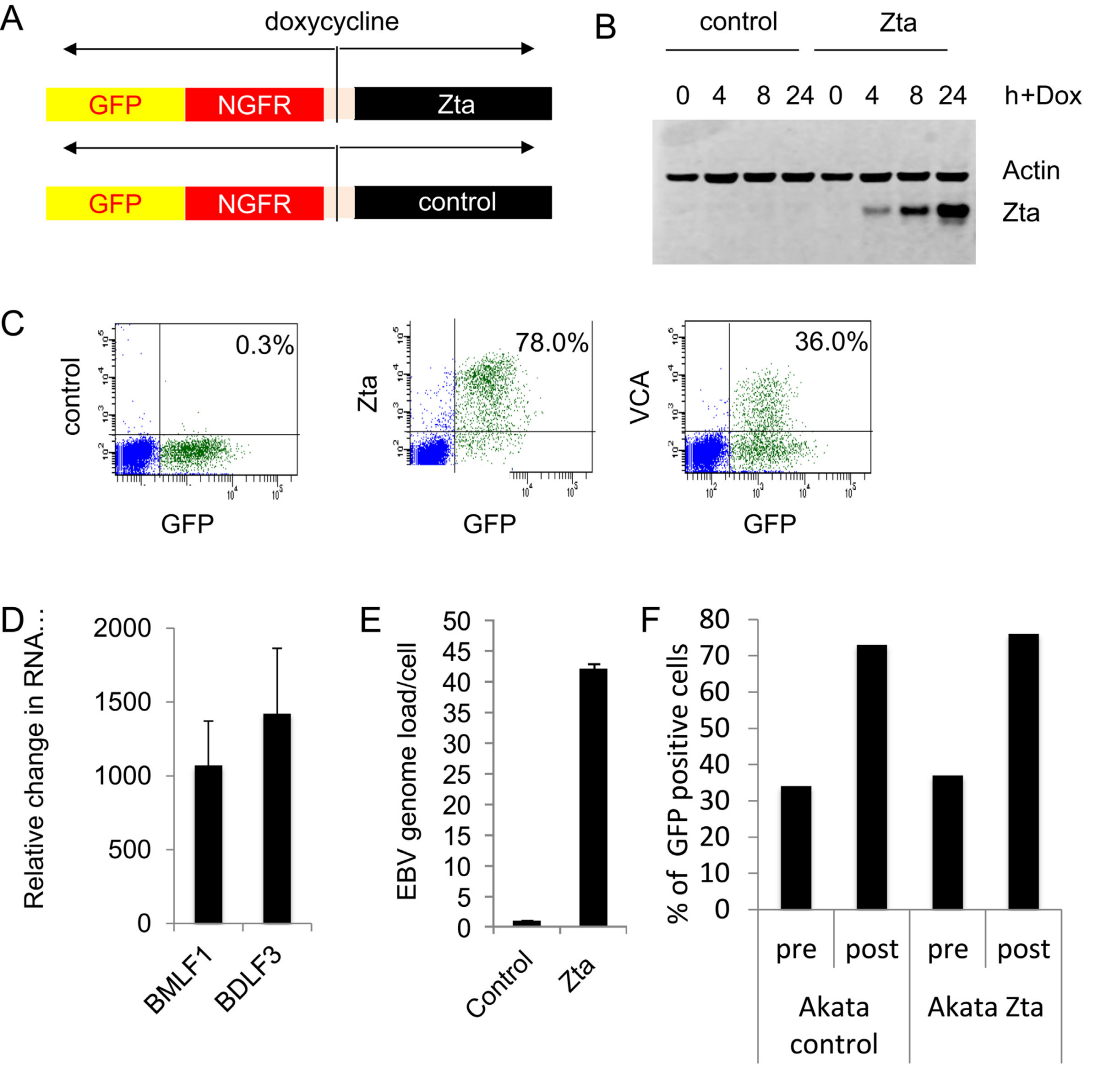
Lytic cycle induction system. (**A**) A schematic diagram of the bi-directional expression vector encoding Zta and is shown. The control vector has the Zta sequence cloned in the opposite direction. (**B**) Protein expression was assessed over 4, 8 and 24 h using immunoblotting for Zta and actin as indicated. (**C**) The expression of Zta and the late lytic cycle protein VCA were determined in Akata Zta cells induced with doxycycline for 24 h using multi-parameter FACS analysis. The data are expressed as the% of the GFP positive cells that also stain for Zta and VCA compared to a control antibody. (**D**) Expression levels of the viral genes BMLF1 and BDLF3 mRNA were assessed using RT-coupled Q-PCR. The relative change in abundance of the mRNA is shown with the error bars relating to triplicate cell induction experiments and standard deviation. (**E**) The relative amount of viral compared to human DNA (viral genome load) was determined by Q-PCR 24 h after doxycycline addition. This is shown with the mean and standard deviation from triplicate assays. (**F**) Cells were induced with doxycycline for 24 h and Zta expressing and control cells were enriched with anti-NGFR coated magnetic beads. The proportion of cells that express GFP is shown for both the pre-enriched and post-enriched populations of cells.

To explore the significance of Zta binding throughout the genome of Akata cells, we wanted to establish whether any of the genes associated with Zta binding sites are transcriptionally regulated during the EBV lytic replication cycle. Previous attempts to investigate changes in cellular gene expression during the EBV lytic cycle have been limited by the low percentage of Akata cells that undergo EBV lytic replication following BCR engagement. To optimize the identification of regulated genes, we introduced an inducible Zta expression vector with a selectable marker gene (32) into Akata cells to allow lytic cycle induction in a high proportion of cells (Figure 3A). In parallel, a cell line was generated with a vector in which the BZLF1 sequences were inserted in the opposite orientation. This cell line was used as a comparator throughout the investigation to control for any impacts of the inducing agent on cells. Addition of doxycycline resulted in the rapid induction of Zta expression (Figure 3B and C) with no associated toxicity (Supplementary Figure S1). This was accompanied by activation of the EBV lytic cycle, as judged by the expression of the late gene VCA (Figure 3C), expression of the viral early gene BMLF1 and late gene BDLF3 (Figure 3D) and replication of the viral genome (Figure 3E). No evidence for lytic replication was observed in the control cells. It is important to note that although a higher percentage of cells express Zta protein in the inducible system compared to BCR stimulation, the abundance of Zta protein is equivalent in the two systems as judged by intracellular staining and FACS analyses (Supplementary Figure S2). Thus, the level of Zta expression induced by doxycycline is physiologically relevant.

**Figure 4.**
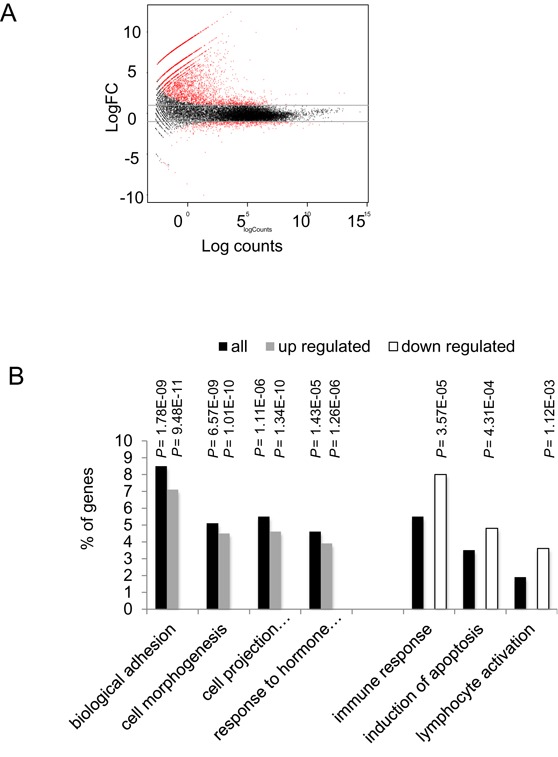
Global transcriptome analysis of gene expression changes reprogrammed during viral replication. (**A**) The expression of cellular genes in two enriched populations of Akata-control and Akata-Zta cells induced with doxycycline for 24 h were assessed using RNA-seq. The distribution of genes with a change in expression of at least two and a false discovery rate (FDR) ≤ 0.01 are shown in red on the MA plot (log total counts versus log fold-change). (**B**) Percentage of genes enriched (Bonferroni-modified *P* ≤ 0.01) for Biological Function categories. Data are shown for all genes (black), those that are up regulated (gray) and those that are down regulated (open).

Populations of control and Zta expressing cells were enriched by selection on anti-NGFR coupled magnetic beads. This generated pools of cells in which greater than 70% expressed the induced genes (Figure 3F and Supplementary Figure S3), in which both the down regulation and the up regulation of gene expression can be determined. This was undertaken for two independently enriched populations of Zta expressing and control cells by sequencing the polyadenylated transcripts (RNA-seq). Differential expression analysis identified 2263 cellular genes whose expression changed >2-fold following induction of the lytic cycle (FDR≤0.01) (Figure 4 and Supplementary Table S2). This included 2242 novel targets and 21 genes that had been identified in previous studies (27,28). Of the genes identified in this analysis, 1679 were up regulated and 584 were down regulated. The genes fell into many functional groups but gene ontology analyses showed that among the up regulated genes, significant enrichment was observed for genes involved in cell adhesion, morphogenesis, projection and response to hormones (Table 2 and Figure 4). Within the down regulated category, there was significant enrichment for genes involved in the immune response, induction of apoptosis and lymphocyte activation (Table 3 and Figure 4).

**Table 2.** Ten most enriched Gene Ontology biological process terms in the set of Zta-up regulated genes

GO term	Accession number	*P* Value	Bonferroni	Benjamini	FDR
Cell projection organization	GO:0030030	3.84E−14	1.34E−10	1.34E−10	7.04E−11
Cell morphogenesis	GO:0000902	5.75E−14	2.01E−10	1.01E−10	1.05E−10
Cellular component morphogenesis	GO:0032989	1.03E−13	3.62E−10	1.21E−10	1.89E−10
Biological adhesion	GO:0022610	1.08E−13	3.79E−10	9.48E−11	1.98E−10
Neuron projection development	GO:0031175	1.62E−13	5.67E−10	1.13E−10	2.97E−10
Cell adhesion	GO:0007155	2.27E−13	7.95E−10	1.33E−10	4.16E−10
Cell morphogenesis involved in differentiation	GO:0000904	7.67E−13	2.69E−09	3.84E−10	1.40E−09
Neuron projection morphogenesis	GO:0048812	1.28E−12	4.49E−09	5.62E−10	2.35E−09
Cell projection morphogenesis	GO:0048858	3.15E−12	1.10E−08	1.23E−09	5.77E−09
Cell morphogenesis involved in neuron differentiation	GO:0048667	7.83E−12	2.74E−08	2.74E−09	1.43E−08

**Table 3.** Ten most enriched Gene Ontology biological process terms in the set of Zta-down regulated genes

GO term	Accession number	*P* Value	Bonferroni	Benjamini	FDR
Immune response	GO:0006955	1.67E−08	3.57E−05	3.57E−05	2.89E−05
Induction of apoptosis	GO:0006917	2.02E−07	4.31E−04	2.16E−04	3.49E−04
Induction of programmed cell death	GO:0012502	2.15E−07	4.59E−04	1.53E−04	3.72E−04
Positive regulation of apoptosis	GO:0043065	2.66E−07	5.68E−04	1.42E−04	4.60E−04
Positive regulation of programmed cell death	GO:0043068	3.06E−07	6.55E−04	1.31E−04	5.30E−04
Positive regulation of cell death	GO:0010942	3.43E−07	7.35E−04	1.23E−04	5.95E−04
Lymphocyte activation	GO:0046649	5.24E−07	1.12E−03	1.60E−04	9.08E−04
Cell activation	GO:0001775	1.14E−06	2.45E−03	3.06E−04	1.98E−03
Regulation of apoptosis	GO:0042981	3.37E−06	7.18E−03	8.01E−04	5.84E−03
Regulation of programmed cell death	GO:0043067	4.38E−06	9.33E−03	9.37E−04	7.59E−03

### Regulation of Zta-associated genes

**Figure 5.**
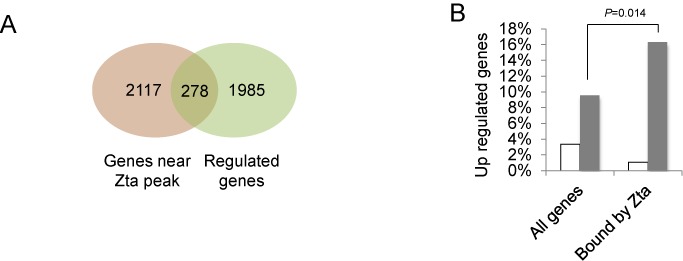
Intersection of ChIP-seq and RNA-seq data. (**A**) The diagram shows the intersection between genes associated with a Zta binding peak (brown) and genes that are regulated during EBV lytic cycle (green). (**B**) The percentage of all cellular genes and the percentage of the sub-set of cellular genes that are associated with Zta binding and up regulated during lytic cycle was compared. The significant increase in the percentage of up regulated genes that are also associated with Zta binding is highlighted (*P* < 0.01, Binomial distribution (cumulative), *n* = 92).

Although the presence of a transcription factor on a gene can be indicative of a regulatory role, it is not definitive: transcription factor binding can occur without discernible changes in gene expression. We therefore asked which of the 2395 cellular genes associated with Zta binding are regulated following Zta induction of the EBV lytic cycle. Of the genes whose expression changed ≥ 2-fold in this experiment, 278 were associated with Zta binding sites and are therefore candidates for direct Zta-mediated regulation (Figure 5A). Of these, 207 genes were up regulated and 71 were down regulated. The presence of one or more Zta binding site correlated with an increased likelihood that a gene was up regulated (*P* = 0.014, Binomial distribution, *n* = 92) (Figure 5B). In contrast, there was no specific enrichment for Zta binding sites in the down regulated genes.

The proportion of genes associated with Zta binding that are demonstrably regulated during EBV lytic replication in BL cells (11.6%) is typical of many cellular transcription factors. Published reports suggest that between 1% and 10% of transcription factor associated genes are transcriptionally altered by expression of the transcription factor (49–52).

**Figure 6.**
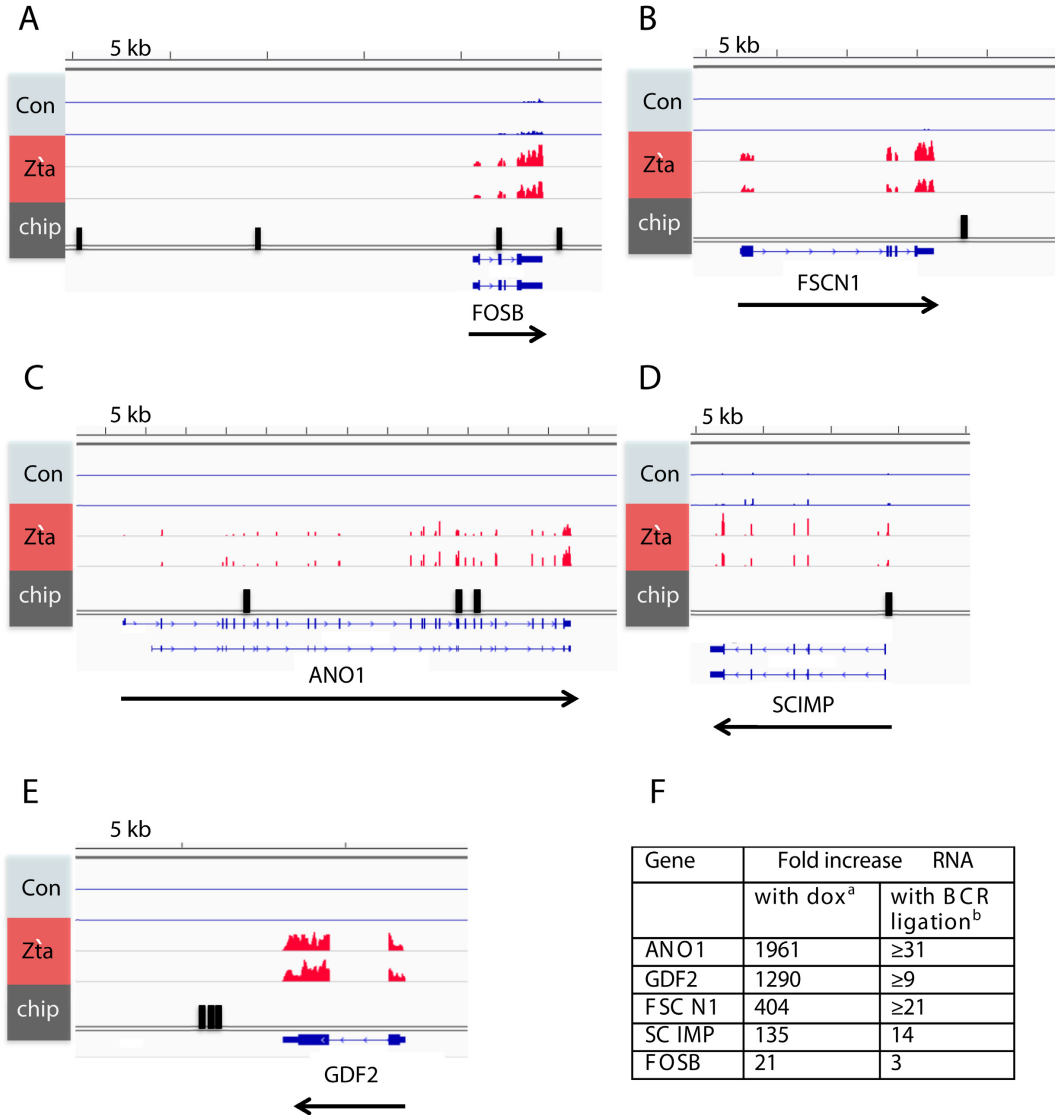
Changes in RNA abundance for five cellular genes after induction of EBV lytic replication cycle in Akata cells. (**A**–**E**) The raw RNA-seq reads from two independent isolations of mRNA for control and Zta expressing cells are aligned to the human genome at *ANO1; GDF2; FSCN1; SCIMP* and *FOSB* loci using the IGV genome browser. A scale bar is shown above each region. The location of the Zta binding sites is shown in black. (**F**) RNA was prepared from Akata cells following crosslinking BCR with anti-IgG for 48 h. Changes in the expression of each gene: *ANO1; GDF2; FSCN1; SCIMP* and *FOSB* were determined using TaqMan RT-PCR. The fold induction data is shown (with BCR ligation, b), together with the fold change from the doxycycline induced Akata Zta and Akata controls cells (with dox, a).

The Zta-bound genes that are also up regulated during EBV lytic cycle, listed in Supplementary Table 3, are the most likely candidates to be regulated directly by Zta and we focused our follow-up analysis on these. We chose a panel of five representative genes in which the Zta binding sites were in different positions relative to the TSS: *SCIMP1, FOSB, ANO1, GDF2* and *FSCN11*. Figure 6 shows the aligned RNA sequence data and the locations of Zta binding sites. For each gene, we obtained appropriate Taq-man probes and quantified the changes in abundance of the RNA in Akata cells following the ligation of BCR by cross-linking with anti-IgG. All of the RNAs showed increased abundance following BCR ligation (Figure 6E), although the effects were difficult to quantify because signals in the unligated cells were below the levels of detection.

### Patterns of Zta binding at regulated genes

**Figure 7.**
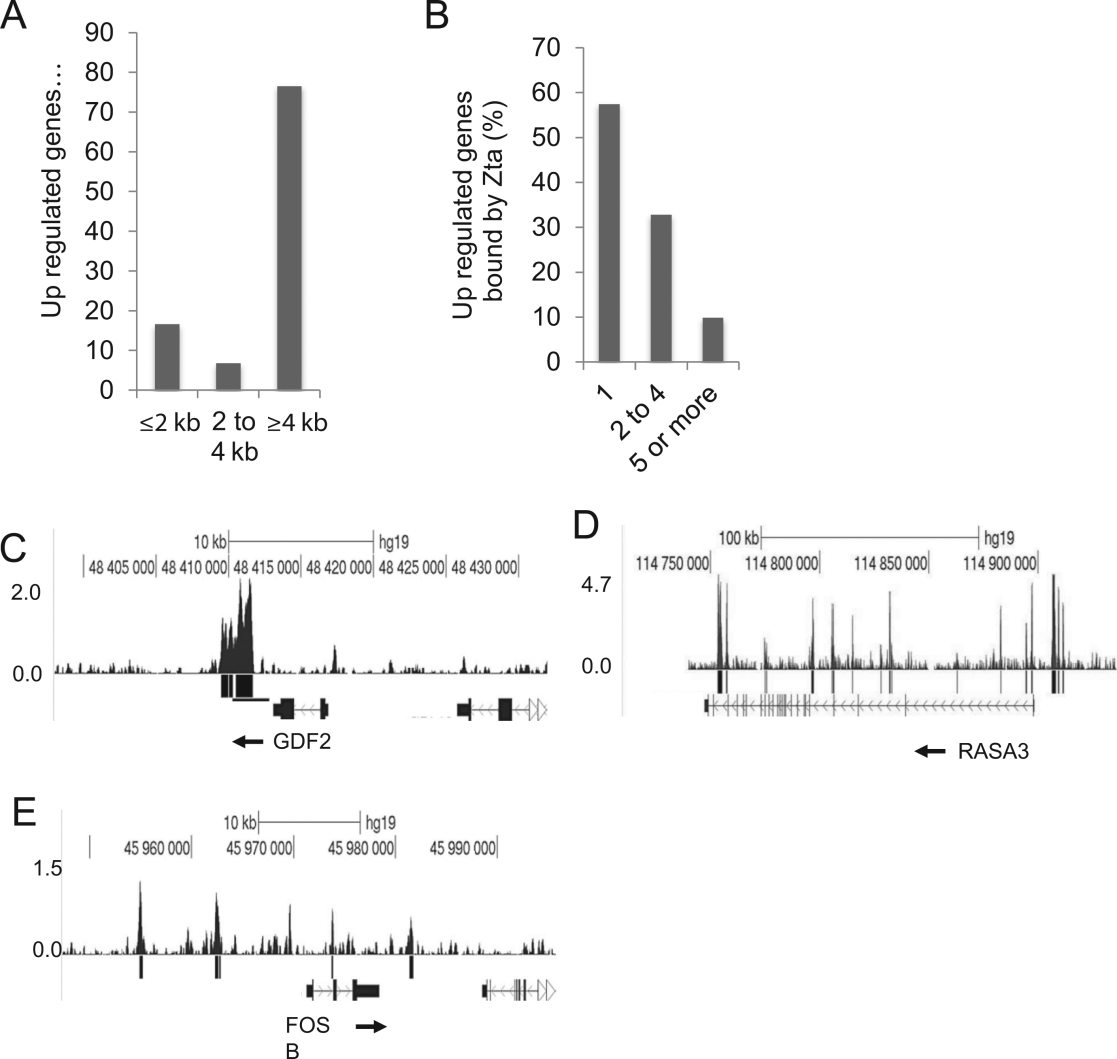
Location of Zta binding sites at up regulated genes. (**A**) The figure shows the percentage of up regulated genes with Zta binding sites within 2 kb, between 2 and 4kb and >4 kb from a TSS. (**B**) The percentage of up regulated genes with 1, 2–4 or >5 Zta binding sites. (**C**) The numbers of sequencing reads from Zta-enriched DNA at the *GDF2* locus (chr10:48 397 968–48 431 996, hg19) are plotted per million background-subtracted total reads and aligned with the human genome. The scale bar and genome location are shown, together with the Zta binding. Beneath this are the peaks identified using MACS (*P* < 10^−7^) and RefSeq gene mapping (UCSC genome browser). (**D**) Similar data for the region surrounding the *RASA3* locus (chr13:114 709 728–114 936 080, hg19). (**E**) Similar data for the region surrounding the *FOSB* locus (chr19:45 947 279–45 995 778 hg19).

Having validated the RNA-seq data at selected targets, we asked whether there was any general correlation between the number and location of Zta binding sites and the observed effects on gene expression at up regulated genes (Figure 7). Although ∼15% of the Zta binding sites were within 2 kb of a TSS, in line with the established model of Zta mediated gene activation of viral promoters, it was clear that the majority (75%) of the Zta binding sites were distal (>4 kb) to the nearest TSS. Approximately half of the up regulated genes associated with a Zta binding site have a single peak whereas the others have two or more (Figure 7).

At some genes, Zta binding was relatively confined as exemplified by the up regulated *GDF2* gene where a cluster of four Zta binding sites was observed at the 3′ end of the gene, 6–8 kb from the transcription start site (Figure 7C). However, at other genes, exemplified by *RASA3* and *FOSB*, Zta binding peaks were found 5′ of the transcription start sites, within intragenic regions and 3′ of the genes, distributed over considerable distances (Figure 7D and E).

### Zta binding sites act as transferable regulatory elements

**Figure 8.**
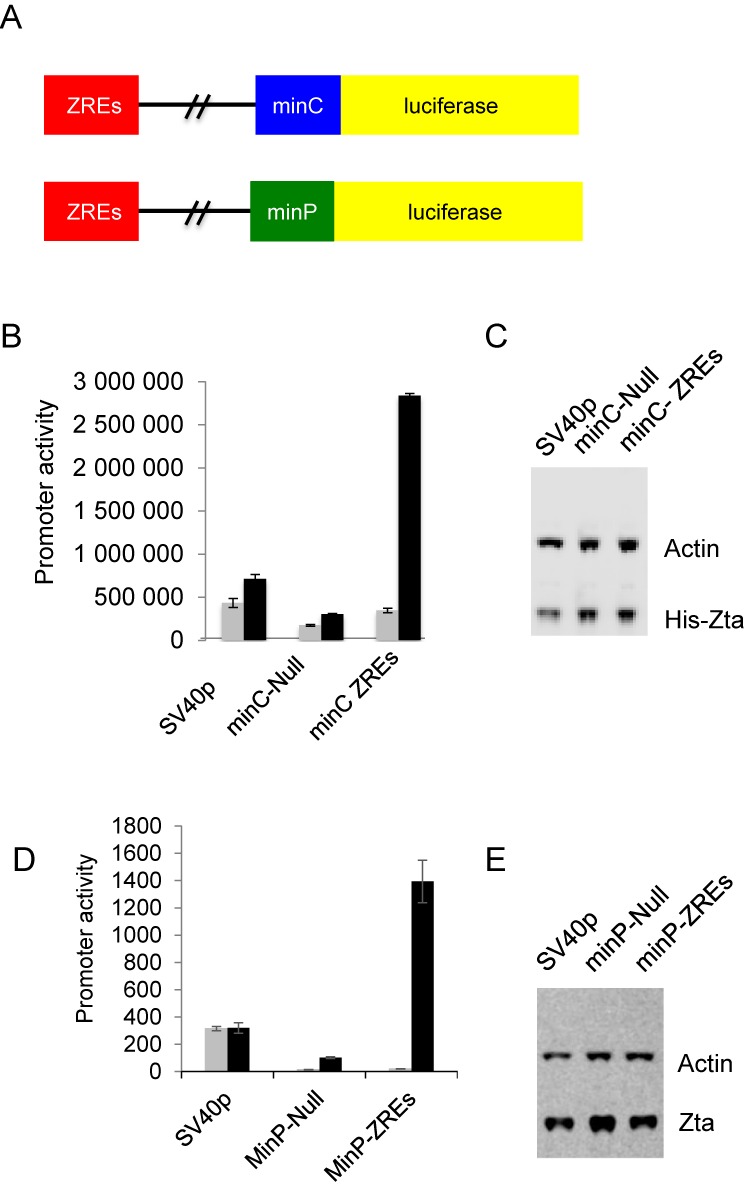
Zta can activate gene expression through a long-range element. (**A**) A synthetic element containing tandem ZREs was cloned 2.2 kb from either of two minimal promoters (minC and minP) in the pGL3 control luciferase reporter plasmid. (**B**) Promoter activities of the pGL3 based plasmids SV40p, minC-Null and minC ZREs in the presence (black) and absence (gray) of His-Zta expression in the EBV negative BL cell line (DG75). The relative luciferase activity is shown as promoter activity together with the standard deviation from three replicates. (**C**) Western blot analysis of Zta and Actin from samples in (B). (**D**) Promoter activity of the minP-Null and minP-ZREs reporters in the presence and absence of His-Zta expression in the EBV negative BL cell line (DG75). The relative luciferase activity is shown as promoter activity together with the standard deviation from three replicates. (**E**) Western blot analysis of Zta and actin from samples in (B).

The ability of Zta to activate transcription by binding to promoter elements is well documented (53) but we are not aware of any previous reports that Zta can act through distal regulatory elements. To gain support for the idea that Zta can also influence transcription from a distance, we generated luciferase reporter constructs in which a tandem array of non CpG ZREs was cloned into a classical enhancer vector, 2.2 kb upstream of one of two minimal promoters (Figure 8A and Supplementary Figure S2). Tandem arrays of ZREs embedded within minimal promoters have been previously used to measure Zta activity (54,55), but action away from a promoter has not been assessed. In the MinC-ZREs reporter, we used a minimal promoter derived from the CIITA gene, which is not regulated by Zta. An alternative reporter, designated MinP-ZREs, was based on the minimal promoter in the commercially sourced pGL4.23 reporter vector (Promega). In each case, we compared the activity of the promoters with and without the ZREs, with an SV40 promoter construct as an additional control (Figure 8B and D). The reporter plasmids were introduced into EBV negative DG75 BL cells, together with a vector encoding Zta or empty vector control. Immunoblotting confirmed that Zta was expressed at equivalent levels in the transfected cell populations (Figure 8C and E). Whereas Zta had a minimal effect on the activity of the MinC and SV40 promoters, the inclusion of the ZRE array resulted in an 8-fold increase in the activity of the MinC construct. Similarly, although Zta appeared able to activate the MinP promoter on its own, the presence of the ZRE array resulted in more substantial activation (75-fold versus 13-fold). The 6-fold differential between these effects is consistent with the impact of the ZRE array on the MinP promoter. Taken together, the data suggest that Zta can regulate gene expression via ZRE elements that are located at a distance from promoters, in a classical enhancer assay.

**Figure 9.**
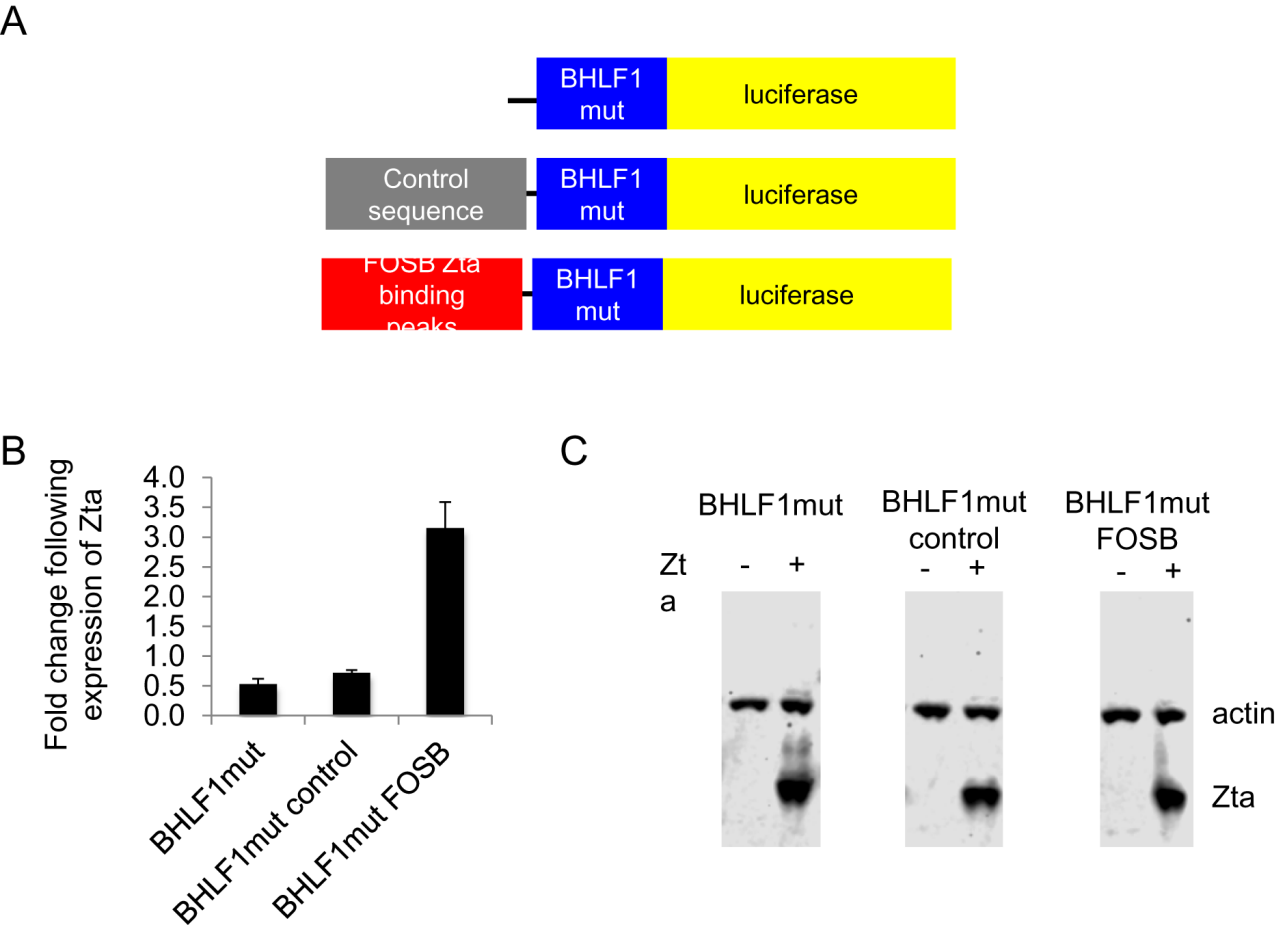
Zta can activate gene expression through binding sites associated with *FOSB*. (**A**) The DNA sequences at three Zta binding sites associated with *FOSB* were cloned upstream from an EBV lytic cycle promoter BHLF1 containing no ZREs (BHLF1mut) to generate BHLF1mut FOSB. A control region from the human genome is also included. (**B**) Promoter activity of BHLF1mut, BHLF1mut control and BHLF1mut FOSB in the presence and absence of His-Zta expression in the EBV negative cell line (DG75). The relative luciferase activity is shown as fold regulation by Zta with the standard deviation from three replicates. (**C**) Western blot analysis of Zta and Actin from samples in (B).

**Figure 10.**
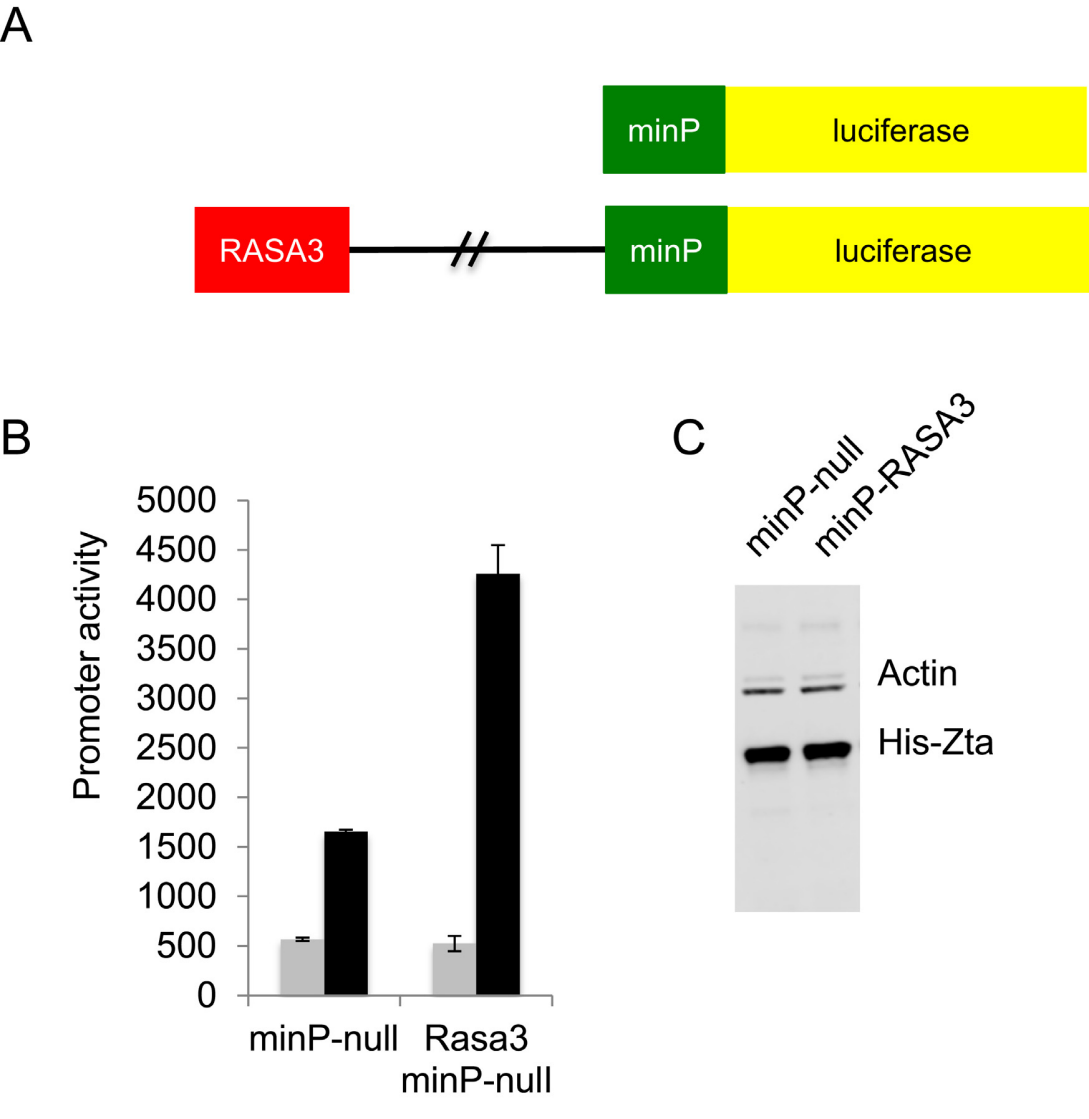
Zta can activate gene expression through binding sites associated with *RASA3*. (**A**) The DNA sequences at seven Zta binding sites associated with the 3′ region of *RASA3* were cloned in the enhancer position >2 kb from the minP promoter to generate minP-RASA3. (**B**) Promoter activity of minP null and RASA3 minP in the presence and absence of His-Zta expression in the EBV negative cell line (DG75). The relative luciferase activity is shown with the standard deviation from three replicates. (**C**) Western blot analysis of Zta and Actin from samples in (**D**).

We then asked whether authentic Zta binding sequences from the cellular targets genes can be transferred onto a heterologous promoter. We chose the *FOSB* and *RASA3* genes as examples. In *FOSB* each of the four non-CPG Zta binding sites was ≥2 kb from the *FOSB* TSS (16 and 9 kb upstream, 2.4 and 10 kb downstream) and the gene is up regulated during the EBV lytic replication cycle (Figure 7E). We cloned the Zta binding sites from −16 and −9 kb upstream from a viral promoter (BHLF1mut) that had been rendered unresponsive to Zta by virtue of mutations in each of its ZREs (Figure 9A). We also included a similar size control region from the cellular genome. The reporter plasmids were transfected into DG75 cells, together with a plasmid encoding Zta or the empty vector control. The presence of the *FOSB* sequences caused a 2.5-fold increase in the response of the promoter to Zta compared to the controls (Figure 9B). Equivalent expression of Zta protein was seen for all reporters (Figure 9C). We therefore conclude that the distal Zta binding sites from the *FOSB* gene are able to confer Zta responsiveness. For *RASA3*, where there are multiple Zta binding sites across a >100 kb region, we focused on elements within seven Zta binding sites from the 3′ end of the *RASA3* gene (≥40 kb from the TSS). These conferred Zta-mediated induction of expression of the minP promoter when cloned into the enhancer position of the PGL3 luciferase reporter vector. The average increase in expression was 2.2-fold over three experiments (Figure 10), which is in line with typical ESC enhancers in similar assays (56).

## DISCUSSION

We identified 2263 cellular genes whose expression is significantly altered within the first 24 h of initiation of the EBV lytic cycle. This was surprising given that a process termed host cell shut-off, driven by the EBV lytic replication cycle gene BGLF5, is reported to promote a global reduction in cellular gene expression during the EBV lytic replication cycle (57–59). The regulation of cellular genes that we observe must therefore occur either in advance of the impact of BGLF5 gene expression or perhaps in spite of it.

It is possible that the cellular genes that are regulated during the EBV lytic replication cycle are specifically targeted to aid the success or the efficiency of viral replication. Indeed, we found a significant enrichment for genes involved in specific biological functions. For example, 190 of the up regulated genes are involved in cell morphogenesis (*P* ≤ 1.0^−10^). We speculate that these genes might facilitate viral assembly or genome encapsulation in the nucleus, the export of immature virions from the nucleus, transport and further assembly within the cytoplasm, or egress from the cell. Among the down regulated group of genes, there was a specific enrichment for genes involved in apoptosis. It is well established that BL cells undergoing EBV lytic replication are protected from apoptosis (60) and the down regulation of the cellular genes identified here might contribute to the observed protection. This suggests that EBV might have evolved a strategy to reprogram cellular gene expression in order to tailor the environment of the cell for optimal viral replication.

During the early stages of the EBV lytic replication cycle, several viral genes, including the transcription factors Zta and Rta and the post-transcriptional regulator BSLF2 + BMRF1, act in concert to regulate the expression of the viral genome (53). In this scenario, it is clear that induction of Zta expression in Akata cells could drive both direct regulation of the host cell transcriptome and instigate indirect regulation of genes through the action of other lytic cycle proteins. It was therefore important to consider which of the 2263 host genes whose expression was altered following Zta activation were associated with a Zta binding peak during the EBV lytic replication cycle. Integration of the data-sets provided 278 cellular genes as candidates for direct Zta-mediated transcriptional regulation.

One of the unexpected findings was that the majority of the Zta binding sites identified in this set of cellular genes are distal to promoters. This is very different from the situation observed in the viral genome where functional ZREs have been shown to lie in very close proximity to transcription start sites (16,20). Indeed, the interaction of Zta with RNA polymerase II accessory proteins is considered to aid the activation of transcription from viral promoters (61–64). The location of the cellular Zta binding sites suggested to us that Zta reprograms the regulation of cellular genes through a different mode of action. In support of this idea, we present two lines of evidence. First, we find that a tandem array of ZREs has enhancer-like activity when located distal to a minimal promoter in a classical enhancer reporter assay. Second, we find that the Zta binding sites from the cellular *FOSB* and *RASA3* genes can transfer Zta-mediated activation onto heterologous promoters. Although the impact is modest, it is in line with the magnitude of regulation recently reported for cellular enhancers in the context of plasmid based reporter assays (56). As the reporter gene regulation occurs in the absence of other EBV gene expression, it supports the hypothesis that Zta is able to directly activate cellular genes through regulatory elements that lie distal to their transcription start sites. This raises the possibility that long-range gene mechanisms act through directing through chromatin looping, in a similar manner to that recently described for the EBV EBNA genes (65,66). In light of the discovery of Zta regulation through distal Zta binding sites, it will be relevant to reconsider the interactions of Zta with the viral genome (16,20), with regards to the potential for regulation of distant promoters.

## SUPPLEMENTARY DATA

Supplementary Data are available at NAR Online.

SUPPLEMENTARY DATA

## ACCESSION NUMBERS

ChIP-sequencing and RNA sequencing data has been deposited in GSE57732.
