# Supplementary material for: Epstein–Barr virus transcription factor Zta acts through distal regulatory elements to directly control cellular gene expression
Source: Nucleic Acids Res. 2015 Mar 16;43(7):3563–77. doi: 10.1093/nar/gkv212 (PMC4402532; doi:10.1093/nar/gkv212)
Supplement: SUPPLEMENTARY DATA [file supp_43_7_3563__index.html]

Epstein–Barr virus transcription factor Zta acts through distal regulatory elements to directly control cellular gene expression — Epstein–Barr virus transcription factor Zta acts through distal regulatory elements to directly control cellular gene expression — SUPPLEMENTARY DATA 

# Epstein–Barr virus transcription factor Zta acts through distal regulatory elements to directly control cellular gene expression

## SUPPLEMENTARY DATA

**Files in this Data Supplement:**

- SUPPLEMENTARY DATA
- SUPPLEMENTARY DATA
