# Supplementary material for: Epstein–Barr virus transcription factor Zta acts through distal regulatory elements to directly control cellular gene expression
Source: Nucleic Acids Res. 2015 Mar 16;43(7):3563–77. doi: 10.1093/nar/gkv212 (PMC4402532; doi:10.1093/nar/gkv212)
Supplement: SUPPLEMENTARY DATA [file supp_gkv212_nar-03402-v-2014-File003.pdf]

Supplementary information for:

Epstein Barr virus transcription factor Zta acts through distal regulatory elements to directly control cellular gene expression

Sharada Ramasubramanyan[1], Kay Osborn1, Rajaei Al-Mohammad[1], Ijiel Barak Naranjo Perez-Fernandez[1], Jianmin Zuo[2], Nicolae Balan[1], Anja Godfrey[1], Harshil Patel[3], Gordon Peters[3], Martin Rowe[2], Richard G. Jenner[4], Alison J. Sinclair[1]*.

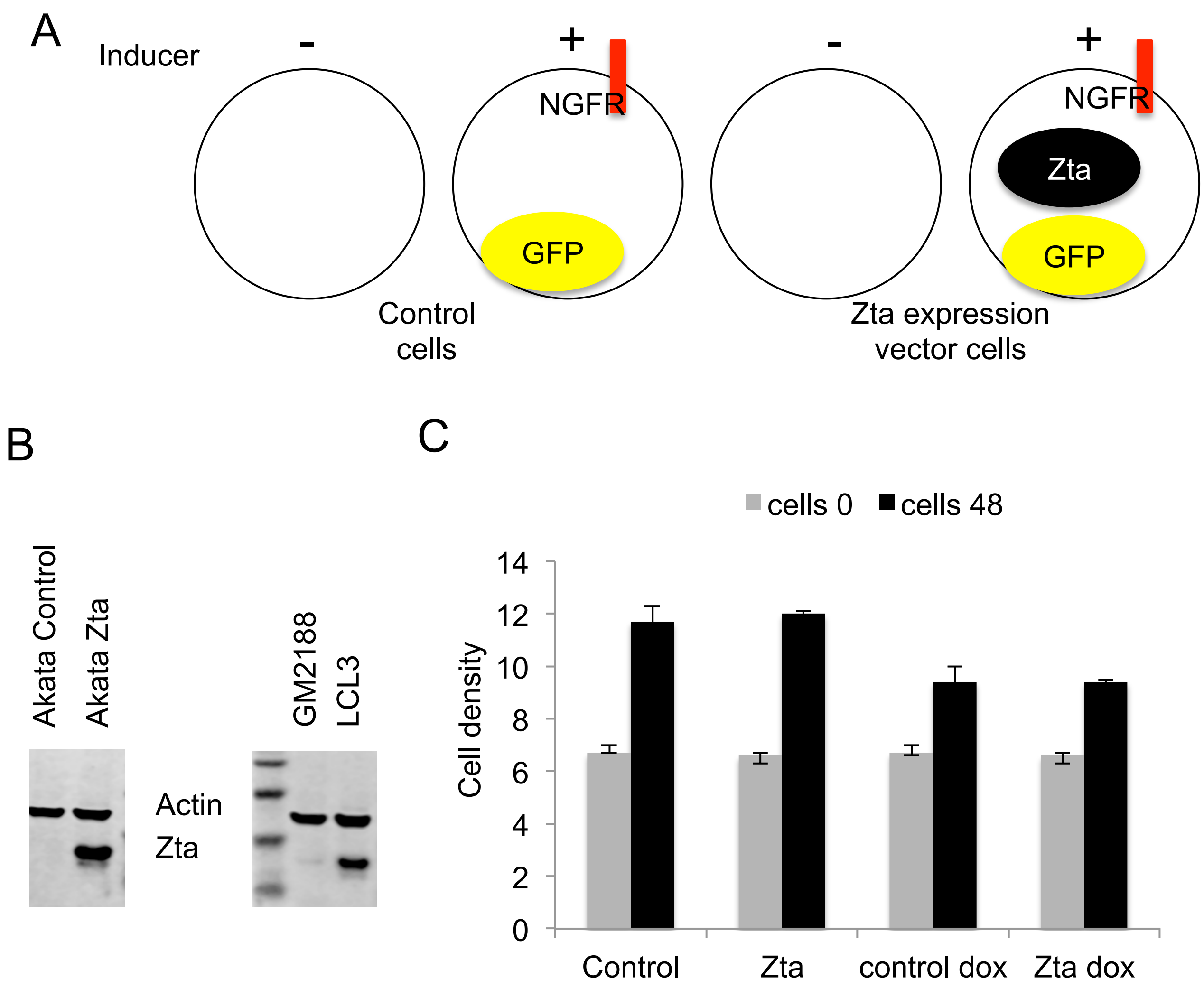

Supporting Figure S1. Generation and characteristics of doxycycline-inducible Akata-Zta and control cells.

A. Expression vectors for Zta and control were introduced into Akata cells and cells expressing the selectable marker CD2 were selected. Doxycycline was added to induced expression of the bi directional promoter and Zta, NGFR and GFP were expressed.
B. Zta and actin proteins were detected by western blot analysis and compared to a tightly latent (GM2188) and a lytic LCL (LCL#3).
C. The viability of the Akata control and Akata Zta cells was assessed using a Trypan Blue exclusion assay after 48 hours growth in the presence or absence of doxycycline.

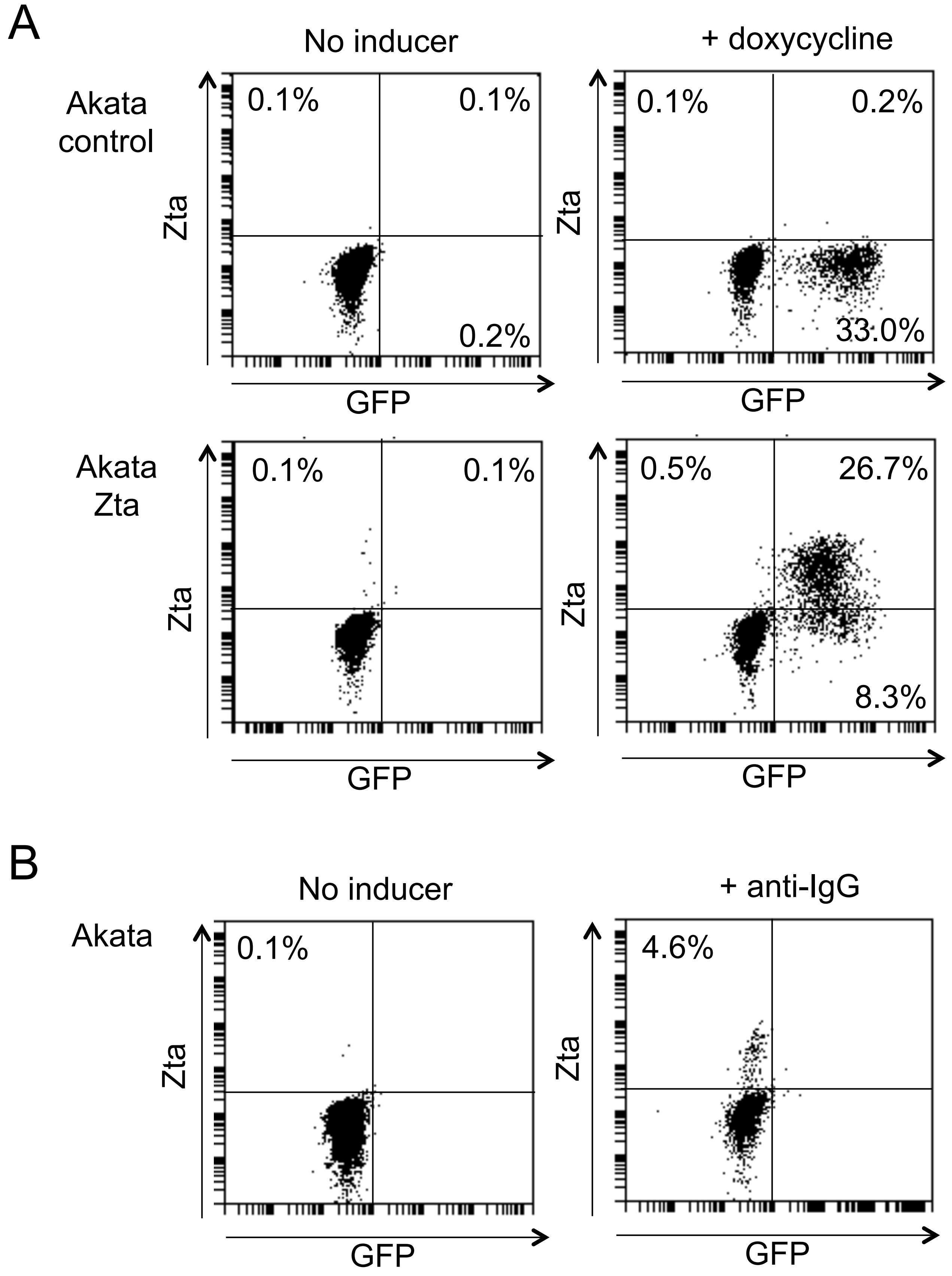

Supporting Figure S2. Comparison of Zta expression after doxycycline induction and BCR ligation.

A. Akata control and Akata Zta cells were exposed or not to doxycycline. The expression of Zta and GFP were detected by multiparameter FACS after intracellular staining for Zta expression.

B. Akata cells were stimulated with and without anti-IgG and the same analysis undertaken.

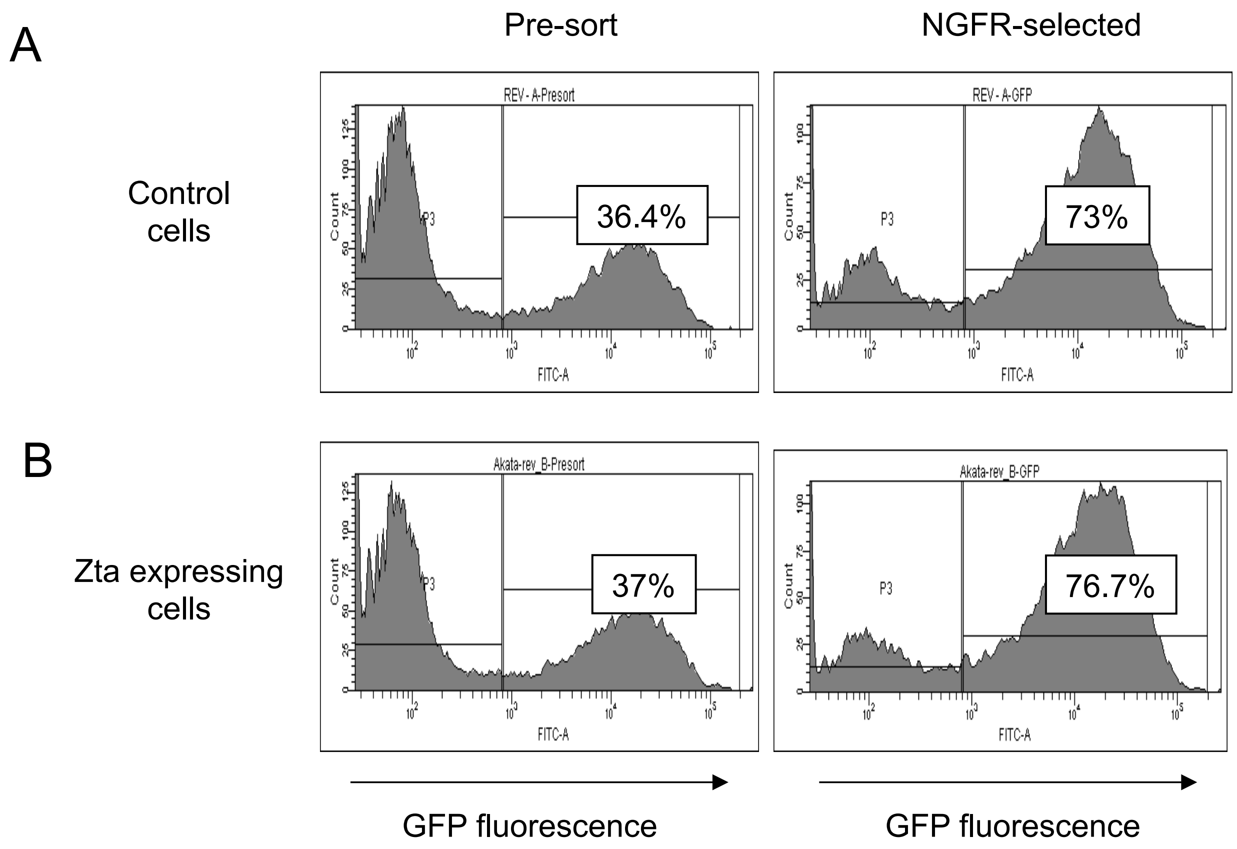

Supporting Figure S3. Enrichment of doxyclyline induced Akata-Zta BL cells.
Cells were induced with doxycycline and analysed for GFP expression by FACS analysis before and after enrichment for NGFR expression.

A. Akata control cells
B. Akata Zta cells.
C. The % of GFP positive cells are shown.

A

CGCCCGGCGGATCCGTCTGCAGAGGCA**TGAGCCA**TGCAC**TTAGCAA**ATTA**TGTGTAA**GTACTG
TGAGCAAGACG**TGTGCAA**TAAGC**TGAGCCA**TGGC**TGTGTCA**ATCGA**TGTGCAA**TGCACATGTG
TCAATGCA**TGAGTCA**TCAT**TGACTAA**TGCTGA**TGTGTCA**GGATA**TGTGTCT**TTAGC**TGAGCAA**
CCTAG**TGGCACA**GCTACC**TGAGTAA**TAGA**GTTGCAA**TACG**TGAGCAA**CTAATC**TGTGTCA**TAC
AG**TGAGCCA**ACTAGTATGTCGACGCGGCCG

B

GGATCCGGGCGAATCTCCGAGCAGAGAGAAGAAATTGAGTGTGTGACAGTGTGAATGGATGTG
ACTATAGGTGAGCAACAGTCGGTGACAATGTGCACATCTGGTATTTCCTGTGTGAGAGCAAAA
CTACAGGACTCAGTGAGTGGTTTCTGTGGTTTGCACTTGTGTATCTGGGTGTATGGGAGTGTG
TAGCCCTGCTGACTGTGTAGATATGAATGTGTGTGCAGTTGTGAGTCTTGCATGTGGTCATCT
ATGTGAAACAATGCCTGTATAAGTACCCATGGATGTGCACGGTTTTGCCTCTAGCCGTGCATT
TCTGGTTCTGTGTGTGTGTGTTTCTGGGACATTCTTCAAGTCCACAACTCTGAGGGTATCACT
GTGAGAGCAGGGGTCCTAGCTCTACCCATTTGTGTGTGTGTGGAGATGTGTAAGTCTATGTAG
ACAAAAGTGTGTGTCATTTACTGTGTTTGGGGTGTGAACACCTATGTGATGTGTTTGCACAAC
TGCACGTGTTTTGTTCTGTGTGTGTGATCGTGTGTTCAAATAAGTCATCTTGTCTAGTCATCA
GGTGCCTGCACAGACAAAGGTGAAAGGTGTCTGCCTGTTGAGATCTGTGGATAGGGTGTATAT
ATGGACATCTCAGCCTGTCTACGTGTGTATCTGTCTCTGTCCTCACGGCAAAAGAGAGGTCGA
C

C

CTTTCTGTCTTCACCAAATTCAGTCCACAGTAAGGAAGTGAAATTAATTTCAGAGGTGTGGGG
AGGGCTTAAGGGAGTGTGGTAAAATTAGAGGGTGTTCAGAAACAGAAATCTGACCGCTTGGGG
CCACCTTGCAGGGAGAGTTTTTTTGATGATCCCTCACTTGTTTCTTTGCATGTTGGCTTAGCT
TGGCGGGCTCCCAACTGGTGACTGGTTAGTGATGAGGCTAGTGATGAGGCTGTGTGCTTCTGA
GCTGGGCATCCGAA

D

GGATAATGGAACCCTATGGATACCTACCTCTAGGCTCCACCCACTAGGTATATCGGGGCGGAG
CCCAATCCTCCCCCTCCTGGTTCAACCCTATGGAGGGGACCCTCCTGAGGCTCCGCCTACCCC
AAATCTCGCGGACCTCTAGCCCCTCCTCCTCTCGTTATCCCAATAGAATAACCTCCAGGTACC
ACCCACCTGGCCCCTTTCCTTAATGAAACCCAACGGGCTAAAACCCCTTTCCTGAATTAACCA
ATAAGAAGCCCCCACTCCCCCCTTTACCTTAAAGTACCCCTTTGAGACCCCAAAAAGAGGATA
AAAGAAGGCGAGCCGGCCCGGCTCGCCAGCGTCGTCCAGACGCTCGGGGGGTGC

E

CGCCCGGCGGATCCGTCTGCAGAGGCA**TTACTCA**TGCAC**TGACTCA**ATTA**TTACTCA**GTACTG
TTACTCAGACG**TTACTCA**TAAGC**TGACACA**TGGC**TGTGTCT**ATCGA**TGTGTCT**TGCACATGTG
TCAATGCA**TGTGTCT**TCAT**TGTGTCT**TGCTGA**TGTGTCT**GGATA**TGTGTCT**TTAGC**TGTGCAA**
CCTAG**TGTGTCT**GCTACC**ACGCACA**TAGA**TGTGCAA**TACG**TGACACA**CTAATC**TGTGTCT**TAC
AG**TGACACA**ACTAGTATGTCGACGCGGCCG

Figure S4. DNA sequence of promoters.

A. The DNA sequence of the tandem ZRE elements is shown with each of the non-CpG ZREs shown in bold.
B. The DNA sequence of the Zta binding sites associated with the FOSB gene are shown (Chr19:45954888-45955223; 45962338-45962553; 45962727-45962858, hg19).
C. The DNA sequence surrounding the *CIITA* promoter (-212 to +54) is shown.
D. The DNA sequence surrounding the viral *BHLF1* promoter is shown with the location of the mutated ZREs underlined. This includes nucleotides 52760-53128 from B95-8 virus sequence.
E. The DNA sequence of the ZREs within seven Zta binding sites associated with the RASA3 3' region are shown (bold).
